# Physiological and biochemical responses of *Limonium tetragonum* to NaCl concentrations in hydroponic solution

**DOI:** 10.3389/fpls.2023.1159625

**Published:** 2023-04-26

**Authors:** Seong-Nam Jang, Min-Ji Kang, Yun Na Kim, Eun Ju Jeong, Kye Man Cho, Jae Gil Yun, Ki-Ho Son

**Affiliations:** ^1^ Department of GreenBio Science, Gyeongsang National University, Jinju, Republic of Korea; ^2^ Department of Plant and Biomaterials Science, Gyeongsang National University, Jinju, Republic of Korea; ^3^ Department of Food Science, Gyeongsang National University, Jinju, Republic of Korea; ^4^ Division of Horticultural Science, Gyeongsang National University, Jinju, Republic of Korea

**Keywords:** halophyte, *Limonium tetragonum*, salt stress, myricetin, hydroponics

## Abstract

**Introduction:**

*Limonium* (L.) *tetragonum* (Thunb.) A. A. Bullock, a halophyte that grows all over the southwest coast of Korea, is a medicinal plant with various pharmacological effects. The salt defense mechanism stimulates the biosynthesis of various secondary metabolites and improves functional substances. In this study, we investigated the optimal NaCl concentration for the growth and enhancement of secondary metabolites in hydroponically grown *L. tetragonum*.

**Methods:**

The seedlings grown for 3 weeks in a hydroponic cultivation system were treated with 0-, 25-, 50-, 75-, and 100-mM NaCl in Hoagland’s nutrient solution for 8 weeks. No significant effect on the growth and chlorophyll fluorescence was observed for the NaCl concentrations below 100-mM.

**Results and discussions:**

The increase in the NaCl concentration resulted in the decrease in the water potential of the *L. tetragonum* leaves. The Na^+^ content accumulated in the aerial part increased rapidly and the content of K^+^, which acts as an antagonist, decreased with the increase in NaCl concentrations in hydroponics. The total amino acid content of *L. tetragonum* decreased compared to the 0-mM NaCl, and most of the amino acid content decreased as the NaCl concentration increased. In contrast, the content of urea, proline (Pro), β-alanine, ornithine, and arginine was increased with an increase in NaCl concentration. The Pro content at 100-mM NaCl accounted for 60% of the total amino acids and was found to be a major osmoregulator as an important component of the salt defense mechanisms. The top five compounds identified in the *L. tetragonum* were classified as flavonoids while the flavanone compound was detected only in the NaCl treatments. A total of four myricetin glycosides were increased in comparison to the 0-mM NaCl. Among the differentially expressed genes, a significantly large change in Gene ontology was seen in the circadian rhythm. NaCl treatment enhanced the flavonoid-based substances of *L. tetragonum*. The optimum NaCl concentration for the enhancement of secondary metabolites of the *L. tetragonum* in the vertical farm-hydroponic cultivation system was 75-mM NaCl.

## 1 Introduction

*Limonium* (L.) *tetragonum* (Thunb.) A. A. Bullock is a biennial, herbaceous, and dicotyledonous plant in the Plumbaginaceae family. It is a halophyte and inhabits the saline environment of Korea’s southwest coast, yet it may also grow in non-saline environments (Ihm et al., 2001). In addition, this plant is physiologically a recretohalophyte with salinity glands and a salt bladder on the leaves, which dilute the plant’s salinity through this structure (Min, 1998). *Limonium tetragonum* is rich in bioactive catechins and flavonoids with anticancerous (Kong et al., 2008), antioxidant (Lee et al., 2011), hepatoprotective (Kim et al., 2015), and antiobesity activities (Kim et al., 2017). The roots and young buds of *L. tetragonum* are widely used as edible vegetables in Korea (Kim et al., 2017). Lee et al. (2011) reported quercetin, myricetin (a natural bioflavonoid), and myricetin glycosides in *L. tetragonum*. Myricetin is also present in various plants such as vegetables, fruit, and medicinal crops (Hertog et al., 1993). Myricetin has the potential to be employed in the treatment of diabetes and cardiovascular disorders due to its antioxidant and pro-oxidative properties (Ong and Khoo, 1997).

Abnormal weather conditions, water scarcity, and reduced available land area due to salinity accumulation negatively impact the production of staple crops such as wheat (Deng et al., 2016). In saline soil, the concentration of ions such as Na^+^ and Cl^−^, which interfere with water absorption of the roots, is high and, thus, has adverse effects on plants such as damage to the chloroplast structure and reduction in plant yield (Soni et al., 2021; Zhu et al., 2022). Halophytes can complete their life cycle at NaCl concentrations above 200 mM (Flowers and Colmer, 2008). To adapt to salt stress, halophytes have morphological, anatomical, and physiological processes (Nikalje et al., 2018). Selective ion uptake by the roots, osmotic regulation, intracellular compartmentalization of toxic ions, and scavenging of reactive oxygen species (ROS) enable survival at high salinity (Meng et al., 2018; Nikalje et al., 2018). Salinity stress is detrimental to most plants, but some may experience eustress. The polyphenol content of safflower (Gengmao et al., 2015), mangroves (Parida et al., 2004), and rapeseed (Falcinelli et al., 2017) was increased with NaCl treatment, suggesting that salt stress at an appropriate concentration may stimulate defense mechanisms and improve physiological activity.

A vertical farm produces crops without time and space restrictions in controlled environmental factors such as light, carbon dioxide, temperature, airflow, humidity, and inorganic nutrients (Son, 2022). The vertical farm has a framework that makes the most of the available area by stacking tall panels (Despommier, 2010). Vertical farms mainly produce leafy vegetables containing various bioactive substances (Zhang et al., 2018; Matysiak and Kowalski, 2019). Leaf vegetables have low economic value but high yields as these crops can be grown year-round on vertical farms. High-value-added crops with several useful compounds are gaining popularity as a solution for the aforementioned problem. Medicinal crops such as *ginseng* (Park et al., 2019) and *nasturtium* (Xu et al., 2021) are grown in vertical farms to be used as high-value-added crops. Efforts have been made to increase economic value as well as the enhancement of secondary metabolites of plants such as *sprout ginseng* (Hwang et al., 2021) and *coriander* (Nguyen et al., 2019). *Limonium tetragonum* is a medicinal plant and can be used as a high-value-added crop. The vertical farm is suitable for its cultivation and the enhancement of secondary metabolites.

The habitat of *L. tetragonum* is flooded once or twice a month, and the seawater flows underground and is greatly affected by NaCl during the growing season (Min, 1998). Although many studies have been done on the physiological efficacy (Bae et al., 2016) and substance analysis (Kim et al., 2017) of *L. tetragonum*, there are few studies on the growth of this plant. Kim (2007) reported that when grown in a nutrient solution environment with different NaCl concentrations in hydroponics, this concentration did not affect the growth of mature *L. tetragonum*. Jeong (2017) reported that the optimal salinity concentration of *L. tetragonum* is 0%–1% and that of phenolic compounds is improved by 1%–2%. Although there are basic studies on the NaCl concentration of *L. tetragonum*, there are few studies on the relationship between NaCl concentration and growth and active substances. Therefore, to investigate the effect of saline stress on the growth, secondary metabolism, and transcriptome of *L. tetragonum*, it was grown at various NaCl concentrations using a hydroponics system on a vertical farm.

## 2 Materials and methods

### 2.1 Plant materials

The seeds of *L. tetragonum* were collected in August 2020 from Gohyeon-myeon, Namhae-gun, South Korea (34°52′07.6″N, 127°53′36.1″E) and stored at 4°C. The seeds were sterilized by immersion in benomyl and thiram wettable powder [benomyl (20%) + thiram (20%)] 0.25% (w/v) for 10 min and subsequently washed three times with sterile water.

### 2.2 Growth conditions

*Limonium tetragonum* seeds were sown in urethane sponge cubes (2 seeds per cell; W × L × H, 25 × 25 × 30 mm) in a cultivation room. After one plant per cell remained, the 3-week-old seedlings were planted in individual pots (ø 45 × height 35 mm). The seedlings per pot and 12 pots per culture panel (36 × 51 cm) were transplanted into a deep flow technique system (W × L × H, 51 × 36 × 7.5 cm). Each treatment was performed in three replicates. In total, 36 plants per treatment were placed in the cultivation room at a temperature of 24°C ± 3°C, relative humidity of 70% ± 10%, and photosynthetic photon flux density of 160 ± 10 µmol m^−2^ s^−1^ [white light-emitting diodes (LEDs), 12 h photoperiod]. Five LED tubes (LED T5, Samsung LED, Korea) were installed per zone, and the white LEDs include red and blue lights (**Figure S1**). The seedlings were given Hoagland’s nutrient solution (HNS; pH 6.5, EC 1.1 dS m^−1^) for 1 week, and then for 8 weeks, NaCl nutrient solution (NNS) of 0-, 25-, 50-, 75-, and 100-mM NaCl concentration prepared by mixing NaCl with HNS was provided (**Tables S1**, **S2**). Before this study, we performed a study and tested a wide range of NaCl concentrations (0-, 50-, 100-, 200-, and 400-mM NaCl). The growth suppression of *L. tetragonum* was observed in NaCl at a concentration of >200 mM (data not published). Excessive NaCl reduces the growth of *L. tetragonum* and is not economically efficient; thus, the purpose of the present study was to focus on the detailed concentrations to improve the growth and functional substances in vertical farms. The pH and electrical conductivity (EC) were measured weekly (**Table S2**) to confirm that the pH was constant, and the NNS was replaced with a fresh nutrient solution every 2 weeks during the experiment to avoid nutrient depletion in the nutrient solution.

### 2.3 Physiological analysis

#### 2.3.1 Growth characteristics

The 8-week-old *L. tetragonum* seedlings were harvested, and 10 plants from each treatment were selected as biological replicates. To investigate the growth characteristics of *L. tetragonum*, fresh weight (FW), the number of leaves, and the total leaf area (LA) of the aerial part and root were measured, and dry weight (DW) was measured after oven-drying (WOF-155, Daihan, Wonju, Korea) at 50°C for 5 days. Samples used for the analysis were ground-dried aerial parts and stored at 4°C until further analysis. The specific leaf weight (SLW) was calculated based on the DW of the leaf per unit LA.


$$SLW=DW(g)/LA(cm^{2})$$

#### 2.3.2 Chlorophyll fluorescence

Chl fluorescence was measured 1 day before harvest in the aerial part treated with different NaCl concentrations for 8 weeks. Four plants were selected from each treatment as biological replicates. The modulated Chl fluorescence parameters were obtained by selecting the broad leaf at the top and dark-adapted for 30 min, and then using a PAM-2100 Chl fluorometer (Heinz Walz GmbH, Effeltrich, Germany) with a saturation pulse (8,000 μmol·m^−2^·s^−1^), before (*F*_0_) and after (*F*_m_) fluorescence were measured for 1 s. The maximum photochemical efficiency of photosystem II (PSII) [*F*_v_/*F*_m_ = (*F*_m_ − *F*_0_)/*F*_m_] was calculated according to the method of Genty et al. (1989).

#### 2.3.3 Leaf water potential

The leaf water potential (LWP) was measured using a dew point water potential meter (WP4C, Meter Environment, Pullman, WA, USA) to investigate the osmotic properties of *L. tetragonum* leaves. The leaves were cleaned by gently rubbing them with distilled water and tissue before cutting them. The widest leaves (diameter, 3 cm) of each plant were collected and then immediately placed in a small steel chamber (diameter, 3.5 cm). The air temperature of the chamber was equilibrated between the inside and outside of the chamber at 24°C for 10 ± 5 min.

#### 2.3.4 Mineral ion content

The mineral ion content in the aerial part of *L. tetragonum* which was grown with various NaCl concentrations was measured in five replicates. Approximately 0.1 g of dried aerial part was digested in 5 ml of 70% HNO_3_ at 125°C for 1.5 h. To stimulate digestion, 7.5 ml of H_2_O_2_ was added to the solution and heated to 123°C for 1 h. HCl (1 ml) was added to the solution again and heated to 200°C for 2 h. The resulting solution was then made up to the same volume (10 ml) using triple distilled water. The solution was filtered through quantitative filter paper (90 mm, Adcantec Co., Japan). The content of 12 elements (Ca, K, Mg, Na, Cd, Cr, Cu, Fe, Mn, Ni, Pb, and Zn) in the solution was determined using an ICP-OES spectrophotometer (Optima 8300 DV; Perkin Elmer, Waltham, MA, USA).

#### 2.3.5 Free amino acids

The amino acid (AA) content was measured in the aerial part using an AA Analyzer(II)_PF (L-8900, Hitachi, Tokyo, Japan). Briefly, HPLC-grade water (5 ml) was added to 0.1 g of dry powdered sample, shaken, and reacted at 60°C in a heating block for 1 h. 5-Sulfosalicylic acid dihydrate 1 ml of 10% was added, left at 4°C for 2 h, and centrifuged for 3 min, and the supernatant was filtered. The supernatant was completely concentrated at 60°C with a vacuum concentrator. Lithium buffer (pH 2.2) was dissolved in a 2-ml solution. This buffer was analyzed after filtering with a membrane filter (pore size, 0.45 µm) in an LC vial, and the analysis was performed in triplicates.

### 2.4 LC-ESI-MS analysis of specific flavonoids in *Limonium tetragonum*

For LC-ESI-MS, an ABSciex 3200 QTRAP mass spectrometer (Applied Biosystems, MDS Sciex, Concord, Canada) was used as the MS system. In LC-ESI-MS, the HPLC system was a Waters ACQUITY UPLC system (Milford, MA, USA). MultiQuant MD software (Version 3.0.1) was used to process the data obtained using UPLC and MS.

LC-ESI-MS HPLC was performed using the method of Lee et al. (2011). The hot air-dried samples were powdered individually using a mortar and pestle and analyzed in five replicates per treatment. After adding 1 ml of methanol to 50 mg of *L. tetragonum* dry powder, ultrasonic extraction was performed for 2 h. Immediately after extraction, 700 μl of the supernatant obtained by separating the precipitate using a centrifuge was taken and filtered with a 0.45-μm PVDF filter. For LC-ESI-MS analysis, the extract was diluted 1,000 times with 50% acetonitrile solvent containing 0.05% formic acid and then injected into the LC.

Five index components were prepared at a concentration of 2,500 ppm using 50% acetonitrile containing 0.05% formic acid and then diluted stepwise. A calibration curve for five index components was prepared using peak area values according to the concentration range of 50 to 500 ppb. The linearity was determined using the correlation coefficient (*r* ^2^).

As mobile phase solvents for LC analysis, 0.1% formic acid DW and 0.1% formic acid MeOH were used, and the analysis conditions are shown in **Table S3**. The column used for the analysis was Waters C18 (100 × 2 mm, 1.7 μm), and the flow rate was set to 0.4 ml/min.

To investigate the changes in secondary metabolite content between the 0-mM NaCl and the NaCl treatments, five major compounds, viz., myricetin-3-O-β-D-galactoside (1), myricetin-3-O-(3″-O-galloyl)-α-L-rhamnoside (2), myricetin-3-O-α-L-rhamnoside (3), myricetin-3-O-(2″-O-galloyl)-α-L-rhamnoside (4), and 5,7,2′,5′-tetrahydroxy flavanone-3-O-β-D-galactoside (5), identified by Lee et al. (2011) in *L. tetragonum* were analyzed. The structures of these five standard compounds were confirmed through NMR analysis after being separated from the extracts of *L. tetragonum* (Lee et al., 2017).

### 2.5 Transcriptome analysis

#### 2.5.1 RNA isolation and sequencing

The *L. tetragonum* leaves treated with different NaCl concentrations for 8 weeks were harvested. Five new leaves with representative growth were selected from each treatment as biological replicates. Trimmomatic (v. 0.39) (Bolger et al., 2014) was used to remove the adapter sequence. Trimming and quality control used the SLIDINGWINDOW, LEADING, and TRAILING options according to the following conditions: 1) window size = 4, 2) mean quality 15, and 3) LEADING, TRAILING ≧3.

#### 2.5.2 Transcriptome *de novo* assembly

*De novo* assembly of cleaned good quality reads was performed using the TRINITY (Grabherr et al., 2011) program. For indexing and mapping, a *de novo* assembled reference file provided by the client (TRINITY software) was utilized.

#### 2.5.3 Enrichment analysis

The gene ontology (GO) of differentially expressed genes (DEGs) was defined using the DAVID tool and the GO consortium database. GO and Kyoto Encyclopedia of Genes and Genomes (KEGG) analyses were performed to obtain more detailed information about DEGs in GO terms (http://geneontology.org/) (Gene Ontology Consortium, 2001) and metabolic pathways (https://www.genome.jp/kegg/). The DEGs in the KEGG pathways and GO analysis were enriched by clusterProfiler R packages. The threshold (padj< 0.01) was determined by the significant enrichment of KEGG pathways and GO terms.

#### 2.5.4 Differential expression analysis

Feature count was performed using the HiSeq-count, and DEGs were identified using the edgeR package tool (Varet et al., 2016). edgeR normalized the data using the truncated mean of the M-values calculated as the weighted average of the log ratios between this test and the baseline after excluding the most expressed genes and the genes with the largest log ratios.

The cutoff criteria established the DEG, which can be “log2 (fold change),” “false discovery rate [adjusted *p*-value (padj)],” and/or “read count.” Default criteria are “|log2(fold change)|>2,” “padj<0.05,” and “read count>1000” for the analyses. DEG enrichment analysis of data sets is performed using tools such as GO, the KEGG pathway, and the STRING database (Szklarczyk et al., 2021).

#### 2.5.5 Annotation

TRINITY IDs were annotated using blastx (e-value ≤ 1e^−10^) against the database Viridiplantae of NCBI NR to understand the function of the gene. InterProScan was used for genome-scale protein function classification because it can perform fast sequence alignment and domain search against selected reference data sets. InterProScan’s settings have been set to default options (Jones et al., 2014).

### 2.6 Statistical analysis

Data were analyzed using Duncan’s multirange test. The one-way analysis of variance was applied using the NaCl concentrations as variables, and mean values were separated by Duncan’s multirange test (*p*< 0.05). All treatments comprised three replicates and each replicate consisted of 10 plants. Statistical analysis was performed using SAS software (version 9.4, SAS Institute Inc., Cary, NC, USA). The mean ± SE values are shown in the tables and figures.

## 3 Results and discussion

### 3.1 Physiological responses

#### 3.1.1 Growth characteristics

The growth of *L. tetragonum* was not affected by the NaCl concentrations (100-mM NaCl) in the nutrient solution (**Table 1** and **Figure S2**). The FW and DW of *L. tetragonum* were the highest at 75-mM NaCl, although there was no significant difference between the 0-mM NaCl and the NaCl treatments. The LA, leaf number, and SLW showed no significant differences between the 0-mM NaCl and the NaCl treatment. Jeong (2017) investigated the changes in plant height, leaf number, FW, and DW of *L. tetragonum* at various NaCl concentrations and reported that NaCl below 171 mM did not affect the growth of *L. tetragonum*. Our results also showed no significant change in growth below 100 mM of NaCl concentrations. Plants are classified into salt-tolerant “halophytes” and salt-tolerant “glycophytes” according to the degree of change in growth in a salinity environment (Flowers and Colmer, 2008). Dicotyledonous halophytes compartmentalize NaCl into vacuoles and store NaCl through the salt bladder and salt gland structures. In addition, halophytes reduce the cytoplasmic NaCl concentration by discharging them out of the leaf (Yuan et al., 2016). According to the method of classifying halophytes by Breckle (1986), *L. tetragonum* is classified as a recretohalophyte with a salt bladder and salt glands on the underside of leaves (Alhaddad et al., 2021). The dicotyledonous halophytes have no significant change in biomass production even at 200 mM of NaCl and have the genetic potential to resist various environmental stresses (O’Leary et al., 1985). The introduction of specific genes related to the resistance of halophytes is being studied as one of the methods to improve the stress tolerance of glycophytes (Himabindu et al., 2016).

**Table 1:** Growth characteristics of *Limonium tetragonum* under different NaCl concentrations.

NaCl (mM)	Weight (g/plant)		Total leaf area (cm^2^)	Number of leaves	SLW (DW g/cm^2^)
	Fresh weight	Dry weight			
0	5.89 ± 0.53	0.88 ± 0.06	96.94 ± 6.14	12.80 ± 0.58	0.09 ± 0.01
25	5.76 ± 0.38	0.88 ± 0.10	94.90 ± 10.56	12.10 ± 0.80	0.08 ± 0.01
50	5.43 ± 0.48	0.82 ± 0.08	92.40 ± 8.99	11.40 ± 0.48	0.07 ± 0.01
75	6.36 ± 0.36	0.94 ± 0.05	102.44 ± 5.68	13.00 ± 0.62	0.09 ± 0.01
100	6.13 ± 0.51	0.89 ± 0.06	100.32 ± 6.97	12.60 ± 0.48	0.08 ± 0.01
Significant	NS	NS	NS	NS	NS

Statistical analysis was performed with Duncan’s multiple range test in the same column. Values are means ± SE (standard error) of 10 replicates.

SLW, a specific leaf weight; NS, non-significant.

#### 3.1.2 Chlorophyll fluorescence

**Figure 1:**
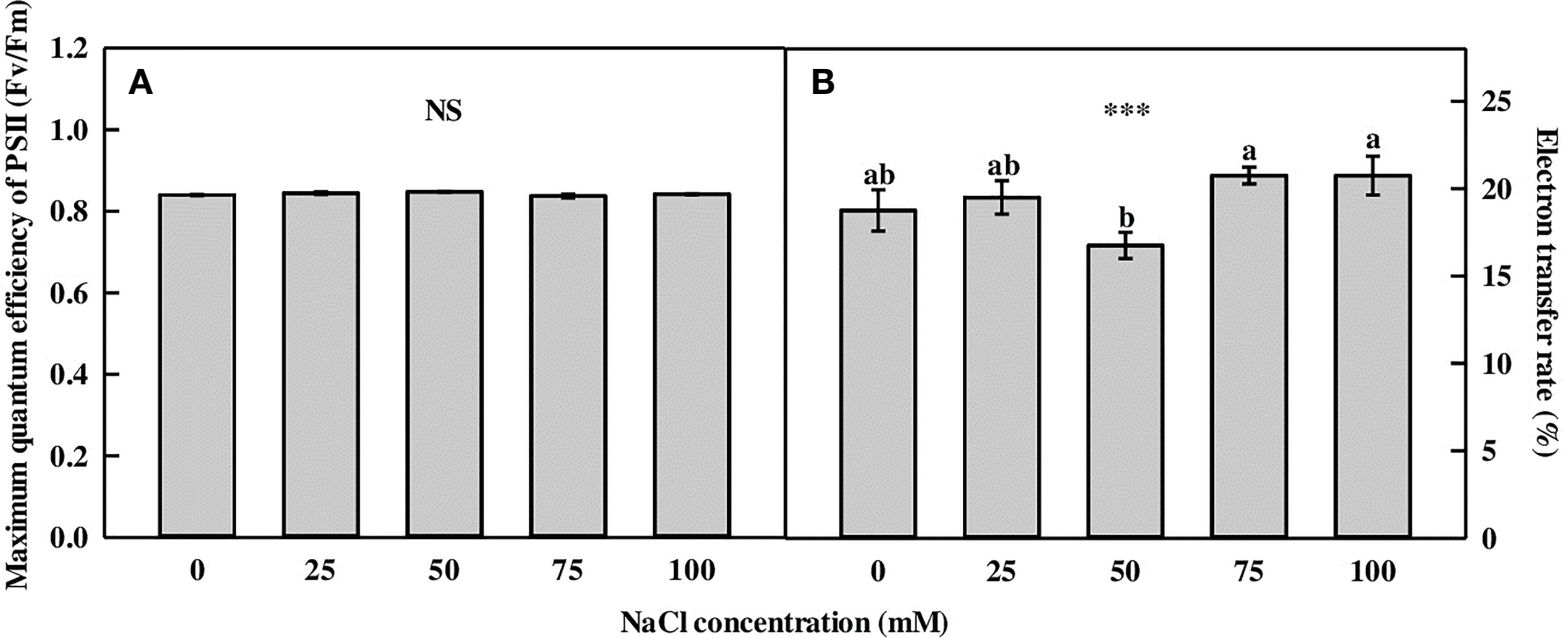
The chlorophyll fluorescence parameters of *Limonium tetragonum* under different NaCl concentrations (*n* = 4). **(A)** Maximum quantum efficiency of PSII (*F*_v_/*F*_m_) and **(B)** electron transfer rate. Statistical analysis was performed with Duncan’s multiple range test. NS, non-significant. ***p = 0.001.

The *F*_v_/*F*_m_ of *L. tetragonum* showed no significant difference between the 0-mM NaCl and the NaCl concentrations (**Figure 1A**). The ratio of *F*_v_/*F*_m_, which reflects a plant’s maximum photosynthetic capacity, is roughly 0.83 in healthy plants (Björkman and Demmig, 1987). The photochemical reaction was not affected by NaCl concentrations below 100 mM. The electron transfer rate (ETR) of *L. tetragonum* was not affected by NaCl treatment (**Figure 1B**). However, the ETR did not decrease at NaCl concentrations of >50 mM; thus, it was considered a temporary plant growth problem when measuring ETR.

Chl fluorescence has been used as an indicator to evaluate the response of plants to environmental stress (Sayed, 2003). Chl fluorescence was used to study the effect of salinity on barley (Belkhodja et al., 1999) and maize (Billah et al., 2017), which decreased at high salinity. However, our results did not show a decrease in Chl fluorescence. The *F*_v_/*F*_m_ of the halophyte *Lycium barbarum* (Wei et al., 2006) and *Panicum turgidum* (Koyro et al., 2013) decreased at high salinity, but not at moderate salinity. This is consistent with the findings of the present study. ETR refers to the number of electrons passing through photosystem II, and high photosynthesis is influenced by ETR (Yao et al., 2017). Since the *F*_v_/*F*_m_ and ETR of the NaCl-treated *L. tetragonum* did not differ from those of the 0-mM NaCl, it was our understanding that the 100-mM NaCl treatment did not affect the photosynthesis of *L. tetragonum*.

#### 3.1.3 Leaf water potential

**Figure 2:**
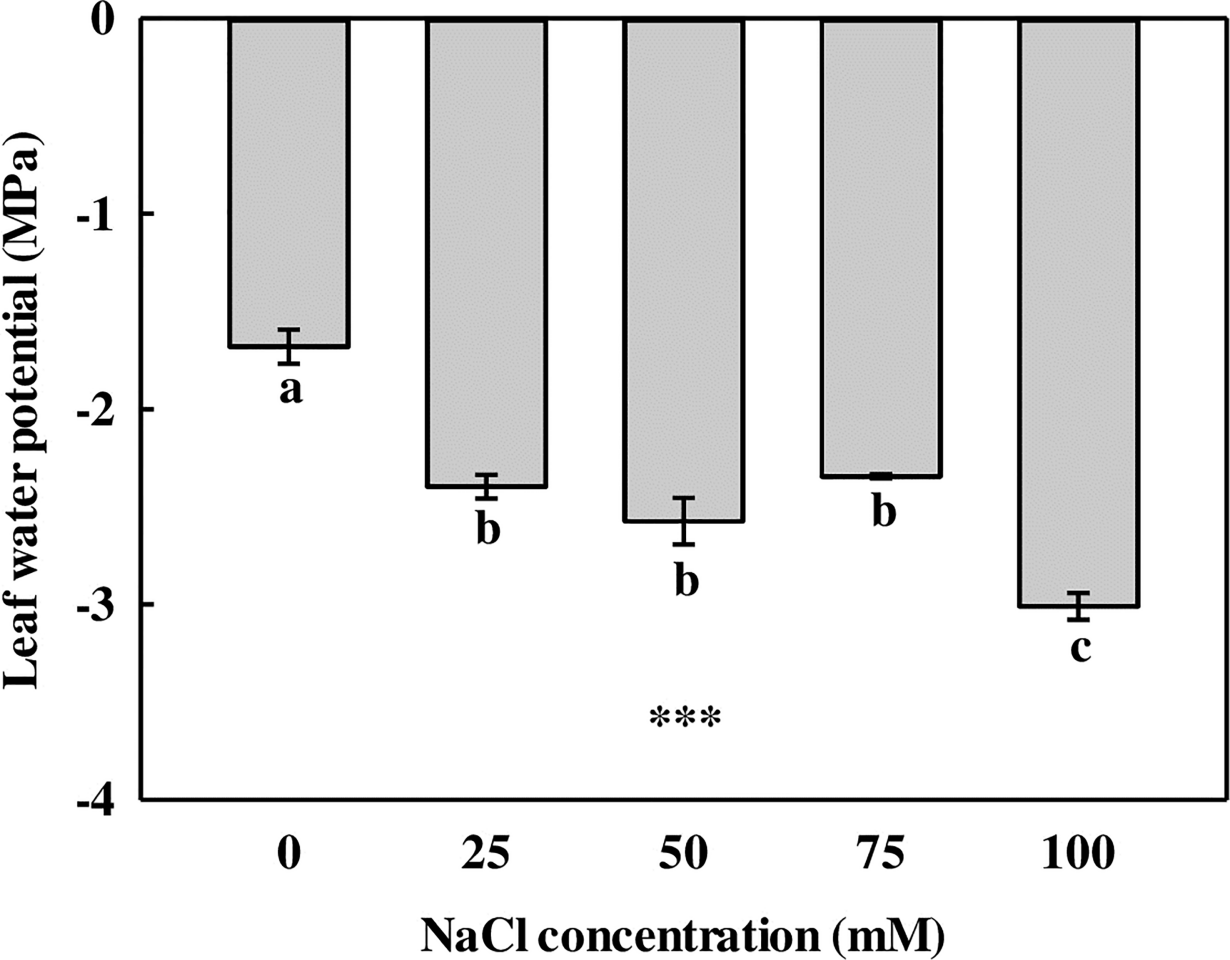
The leaf water potential of *Limonium tetragonum* under different NaCl concentrations (*n* = 4). Statistical analysis was performed with Duncan’s multiple range test. ***p = 0.001.

The leaves showed a significant difference in LWP in comparison with the 0-mM NaCl (**Figure 2**). The LWP of *L. tetragonum* was significantly higher in the 0-mM NaCl (−1.68 MPa) than in the NaCl treatments. The LWP of *L. tetragonum* grown under 25-, 50-, and 75-mM NaCl showed no significant difference, and the LWP of 100 mM of NaCl showed the lowest value (−3.01 MPa) compared with the other treatments. Water potential is a numerical value of the potential energy of water with respect to the content of the solute. The lower value of the water potential indicates a higher concentration of the solute compared with the solvent (Zhu et al., 2022). The amount of solutes accumulated in the leaves of *L. tetragonum* increased in the NaCl treatment in comparison with the 0-mM NaCl. The amount of NaCl accumulated in the leaves of *L. tetragonum* showed a tendency to increase at 100 mM compared with the NaCl accumulated at 75 mM. Salinity significantly affects the water potential of plants and soil. Excessive accumulation of salts in the soil increases the concentration of solutes, lowering the water potential. Water potential gradient differences between soil and plants reduce water uptake in plants such as tomatoes (Tanveer et al., 2020) and maize (Ali et al., 2021), which is the osmosis action of salt stress. However, dicotyledonous halophytes adapt to this osmotic stress by adjusting the NaCl concentration. Although a reduction in LWP was observed, growth remained unaffected with the increase in NaCl concentration; this indicated that the leaves of *L. tetragonum* were osmotically adjusted to survive in a saline environment.

#### 3.1.4 Mineral ion content

##### 3.1.4.1 Macronutrients and Na^+^

The content of monovalent ions in the aerial part of *L. tetragonum* was changed significantly in accordance with the NaCl concentrations (**Table 2**). The K^+^ content was the highest in the 0-mM NaCl at 47.25 mg, and the content decreased significantly as the NaCl concentration increased. The 75-mM NaCl had the lowest K^+^ content (55.3% less in comparison with the 0-mM NaCl) among all the NaCl treatments. NaCl treatment significantly reduced the K^+^ content in the leaves of *L. tetragonum*. The Na^+^ content increased proportionally to the NaCl concentration, and there was a significant difference among all the NaCl concentrations. The Na^+^ content in the 25- and 50-mM NaCl increased approximately five times compared with the 0-mM NaCl and approximately 50 times at the 100-mM NaCl. A significant difference in K^+^/Na^+^ between different NaCl concentrations was observed. It showed the highest value at 59.55 mg in the 0-mM NaCl and was decreased by approximately 58 times or more in the NaCl treatments. The highest amount of Ca^2+^ (10.44 mg) and Mg^2+^ (9.9 mg) was observed at 25 mM.

**Table 2:** Mineral ions and Na^+^ content of *Limonium tetragonum* under different NaCl concentrations.

NaCl concentration (mM)	0	25	50	75	100	Significant
Macronutrient (mg/g DW)						
Ca	9.86 ± 0.06 b	10.44 ± 0.02 a	9.67 ± 0.10 b	6.68 ± 0.10 d	8.35 ± 0.08 c	***
K	47.25 ± 0.22 a	26.38 ± 0.07 b	26.68 ± 0.20 b	21.09 ± 0.26 d	21.84 ± 0.16 c	***
Mg	8.61 ± 0.04 b	9.90 ± 0.02 a	8.70 ± 0.07 b	7.13 ± 0.09 d	7.54 ± 0.06 c	***
Na	0.80 ± 0.03 e	23.40 ± 0.06 d	25.94 ± 0.11 c	40.30 ± 0.33 b	45.15 ± 0.21 a	***
K/Na	59.55 ± 2.00 a	1.13 ± 0.01 b	1.03 ± 0.01 b	0.53 ± 0.01 b	0.49 ± 0.01 b	***
Micronutrient (µg/g DW)						
Cd	N.D.	N.D.	N.D.	N.D.	N.D.	–
Cr	1.00 ± 0.08 c	0.90 ± 0.04 c	3.65 ± 0.09 a	0.95 ± 0.07 c	1.60 ± 0.04 b	***
Cu	15.45 ± 5.17 a	12.70 ± 1.02 a	11.13 ± 0.79 a	10.60 ± 0.52 a	10.35 ± 0.47 a	NS
Fe	137.8 ± 2.10 c	129.40 ± 0.43 d	141.90 ± 0.42 b	157.20 ± 0.78 a	112.70 ± 0.51 e	***
Mn	73.35 ± 0.38 a	37.30 ± 0.12 d	40.00 ± 0.12 c	37.80 ± 0.17 d	54.45 ± 0.34 b	***
Ni	N.D.	N.D.	N.D.	N.D.	N.D.	–
Pb	6.35 ± 0.48 a	6.50 ± 0.24 a	7.50 ± 0.28 a	6.25 ± 0.31 a	6.35 ± 0.15 a	NS
Zn	19.05 ± 0.17 a	18.15 ± 0.09 b	19.35 ± 0.04 a	15.90 ± 0.04 c	19.00 ± 0.09 a	***

Statistical analysis was performed with Duncan’s multiple range test in the same row. Values are means ± SE of five replicates.

N.D., non-detected; NS, non-significant..

***p = 0.001.

K^+^ is an essential ion in plant physiology, which plays a vital role in osmotic pressure regulation, maintenance of swelling, and protein synthesis (Munns and Tester, 2008). However, absorption is inhibited by Na^+^, leading to a decrease in the concentration of K^+^ in plants (Qu et al., 2012), which leads to stomata closure and reduced photosynthetic rate and reduces plant yield (Siegel et al., 2009). The increase in NaCl concentration in the succulent stem of the perennial halophyte *Sarcocornia quinqueflora* decreased the K^+^ content. The K^+^ content showed a positive correlation with ascorbic acid and soluble sugar (Ahmed et al., 2021). The ability of the halophyte to accumulate salt varies from species to species. The NaCl content of the aerial part of *T. decumbens* increased approximately 7.9 times compared with the 0-mM NaCl (Sogoni et al., 2021). Accordingly, *L. tetragonum* showed excellent salt accumulation ability among halophytes. High concentrations of Na^+^ interfere with the synthesis of K^+^ transporter-associated KUP-HAK transporters, which leads to a decrease in K^+^ and, consequently, a decrease in K^+^/Na^+^ (Santa-María et al., 1997). However, halophytes have salt tolerance by self-regulating the NaCl concentration in the plant due to special organs (Yokoi et al., 2002). In halophytes, K^+^/Na^+^ is used as an indirect indicator of salt tolerance (Greenway and Munns, 1980). K^+^/Na^+^ decreased approximately 58-fold in the NaCl treatment in comparison with the 0-mM NaCl, indicating high salt tolerance of *L. tetragonum*. The leaves of *L. tetragonum* showed comparable accumulation tendencies for Ca^2+^ and Mg^2+^. The *Limonium* species showed a common decrease in Ca^2+^ and Mg^2+^ in all plants due to high NaCl concentrations, which was in line with our study (Al Hassan et al., 2017). Ca^2+^ is a substance involved in salinity and osmotic signaling that protects plants from ROS (Knight et al., 1997). Although 100 mM of NaCl reduced Ca^2+^ and K^+^ in the leaves of *L. tetragonum*, it does not affect plant growth due to the increased utilization efficiency of these minerals (Ghnaya et al., 2007).

##### 3.1.4.2 Micronutrients

The salinity changed the content of trace elements in the aerial part of *L. tetragonum* (**Table 2**). Cr and Fe increased in the NaCl treatments than in the 0-mM NaCl, and Mn and Zn^2+^ decreased in the NaCl treatments. Cu^2+^ and Pb^2+^ showed no significant change between the 0-mM NaCl and the NaCl treatment, and neither Cd^2+^ nor Ni was detected. Fe showed the lowest value at 100 mM of NaCl, and it decreased by 20% compared with the 0-mM NaCl. Mn showed the lowest content at 25 and 75 mM of NaCl and decreased by approximately 65% in comparison with the 0-mM NaCl. Zn^2+^ showed the lowest content at 75 mM of NaCl and decreased by 18% in comparison with the 0-mM NaCl.

Micronutrients such as iron, zinc, or copper are necessary nutrients for all living organisms (Waters and Sankaran, 2011). Depending on the type of plant, the effects of salt stress on micronutrients varied. Appropriate NaCl concentration increased micronutrients in zucchini (Víllora et al., 2000).

### 3.2 Free amino acids

The free AAs accumulated in the aerial part of *L. tetragonum* showed apparent differences according to the NaCl concentrations (**Table 3**). The free AA content changed proportionally with the NaCl concentration. Treating *L. tetragonum* with NaCl led to an increase in the non-essential AAs—urea, proline (Pro), β-alanine (β-Ala), ornithine (Orn), and arginine (Arg)—and a decrease in the remaining non-essential AAs. There was an overall decrease in essential AAs in NaCl treatment. Alanine accounted for 25% of the non-essential AAs and showed the highest content in the 0-mM NaCl. In contrast, Pro accounted for 60% of the total non-essential AAs in the NaCl treatment, and the total non-essential AA content of the NaCl treatment decreased compared with that of the 0-mM NaCl. The total AA content was maintained due to Pro. The accumulated Pro in the aerial part of *L. tetragonum* increased rapidly as the NaCl concentration increased and was found to be a major AA constituting most of the total AAs.

**Table 3:** Free amino acids of *Limonium tetragonum* under different NaCl concentrations.

NaCl concentration (mM)	0	25	50	75	100	Sig
General amino acids (µg/g DW)						
Taurine	N.D.	5.3 ± 1.4 b	N.D.	6.7 ± 0.9 ab	10.3 ± 0.3 a	**^w^
Urea	20.5 ± 10.3 b	185.3 ± 10.5 a	141 ± 7.2 a	167.4 ± 10.1 a	157.5 ± 2.7 a	***
Proline	283.9 ± 0.6 d	560.6 ± 3.1 b	490.4 ± 1.6 c	951.7 ± 6.5 a	939.0 ± 2.9 a	***
Aspartic acid	91.6 ± 5.5 a	71.4 ± 1.9 b	69.8 ± 0.6 b	59.8 ± 2.2 bc	50.0 ± 0.4 c	***
Glutamic acid	219.4 ± 9.2 a	150.3 ± 1.2 b	156.1 ± 0.4 b	166.8 ± 3.6 b	158.6 ± 1.4 b	***
Aminoadipic acid	48.6 ± 2.1 a	33.7 ± 0.9 b	29.1 ± 1.2 bc	25.1 ± 2.8 c	4.9 ± 0.7 d	***
Glycine	9.2 ± 0.2 a	7.5 ± 0.1 c	8.2 ± 0.1 b	6.6 ± 0.1 d	6.9 ± 0.1 d	***
Alanine	430.7 ± 7.6 a	275.3 ± 4.1 b	278.6 ± 3.1 b	204.9 ± 0.5 c	206.4 ± 1.4 c	***
Citrulline	N.D.	5.5 ± 0.1 b	9.0 ± 0.4 b	7.5 ± 0.1 b	17.0 ± 1.6 a	***
Cystine	100.4 ± 5 a	97.5 ± 0.6 ab	89.2 ± 1.2 b	79.5 ± 0.9 c	63.5 ± 1.6 d	***
Cystathionine	39.0 ± 1.0 a	30.7 ± 0.8 a	27.9 ± 1.8 a	28.1 ± 0.7 a	15.5 ± 3.7 b	***
Tyrosine	64.4 ± 0.8 a	53.9 ± 0.4 b	26.9 ± 0.7 d	48.6 ± 0.5 c	27.6 ± 1.7 d	***
β-Alanine	6.4 ± 0.1 c	23.5 ± 0.2 b	22.7 ± 0.2 b	22.6 ± 0.1 b	25.9 ± 0.3 a	***
Ornithine	N.D.	1.1 ± 0.1 c	1.7 ± 0.1 b	3.2 ± 0.1 a	3.2 ± 0.1 a	***
Arginine	149.5 ± 2.4 c	157.1 ± 2.1 b	135.1 ± 2 d	154.7 ± 0.2 bc	167.6 ± 1.4 a	***
Totals	1,702.1 ± 21 a	1,574.5 ± 13.9 bc	1,488.2 ± 13.9 c	1,589.7 ± 16.2 b	1,551.5 ± 14.4 bc	***
Essential amino acids (µg/g DW)						
Threonine	100.4 ± 4.9 a	67.6 ± 0.6 b	64.9 ± 0.5 b	51.8 ± 0.4 c	38.8 ± 0.5 d	***
Valine	72.8 ± 2.1 a	74.0 ± 1.3 a	58.0 ± 0.5 b	59.7 ± 0.6 b	51.5 ± 1.2 c	***
Methionine	24.4 ± 0.4 a	21.9 ± 0.2 ab	19.8 ± 0.9 b	20.7 ± 0.7 ab	12.2 ± 0.8 c	***
Isoleucine	45.4 ± 0.6 a	34.5 ± 0.2 b	34.5 ± 1.9 b	30.5 ± 0.4 b	9.3 ± 0.1 c	***
Leucine	58.4 ± 0.2 a	42.5 ± 0.1 b	39.6 ± 1.8 bc	35.6 ± 0.2 c	18.9 ± 0.2 d	***
Phenylalanine	73.9 ± 1.1 a	63.5 ± 0.2 b	33.1 ± 0.6 e	58.4 ± 0.5 c	52.3 ± 1.3 d	***
Lysine	38.5 ± 0.4 a	28.9 ± 0.3 b	29.6 ± 0.3 b	24.8 ± 0.1 d	26.3 ± 0.1 c	***
Histidine	15.0 ± 0.3 a	10.3 ± 0.1 b	10.5 ± 0.1 b	8.6 ± 0.1 d	9.2 ± 0.1 c	***
Totals	428.3 ± 9.2 a	342.9 ± 1.0 b	289.5 ± 2.9 c	289.6 ± 1.3 c	218.1 ± 0.6 d	***
Total amino acids (µg/g DW)						
Ammonia	63.8 ± 0.6 a	41.9 ± 0.2 b	42.4 ± 0.5 b	34.5 ± 0.1 c	30.8 ± 0.8 d	***

Statistical analysis was performed with Duncan’s multiple range test in the same row. Values are means ± SE of three replicates.

Sig, significant value; N.D., non-detected.

**p = 0.05; ***p = 0.001.

Various AAs such as proline, glutamic acid, glycine, alanine, and arginine are used as indicators of plant stress tolerance (Younis et al., 2009). Pro is an AA that controls osmosis together with glycine, betaine, and total soluble sugar. Pro acts as an antioxidant and is involved in the antioxidant system of halophytes. Pro is an osmotic substance that is synthesized to stabilize the intracellular osmotic balance. Most halophytes accumulate Na^+^ in vacuoles in cases of excessive salt absorption. Increased solute concentration in the vacuole leads to an increase in the Pro content to adjust the osmotic pressure outside the vacuole (Szabados and Savouré, 2010). Similarly, in this study, the Pro content of the leaves increased with increasing NaCl concentration. Salt stress is accompanied by water stress, and Pro is easily synthesized from glutamate, ornithine, and arginine in *Cyclotella cryptica* (Liu and Hellebust, 1976). In the salt-treated *L. tetragonum*, glutamate appears to be mostly converted to Pro and reduced. Arg is one of the AAs that serves as a precursor to proteins as well as nitric oxide, urea, proline, and glutamate (Wu and Morris, 1998). Orn is the major metabolite of Arg in the urea cycle and shares many pharmacological effects with Arg (Endo et al., 2004). Orn has a wound-healing effect (Shi et al., 2002). A small amount of Orn was detected in the salt-treated *L. tetragonum*, but both Orn and Arg were increased compared with the 0-mM NaCl, suggesting the possibility that salt stress can improve the wound-healing effect of *L. tetragonum*. β-Ala is present in all living organisms and is required for plant growth as it is a precursor to vitamin B_5_ and pantothenate (Raman and Rathinasabapathi, 2004). In most plants of the Plumbaginaceae family, β-Ala is converted to β-Ala betaine, an osmoprotectant, which is involved in salt tolerance (Rathinasabapathi et al., 2001; Raman and Rathinasabapathi, 2003). The content of β-Ala was increased by various stresses (Parthasarathy et al, 2019). The β-Ala content increases along with other non-essential AAs under slight salt stress caused by low and medium concentrations of NaCl.

### 3.3 Contents of specific flavonoid compounds

**Figure 3:**
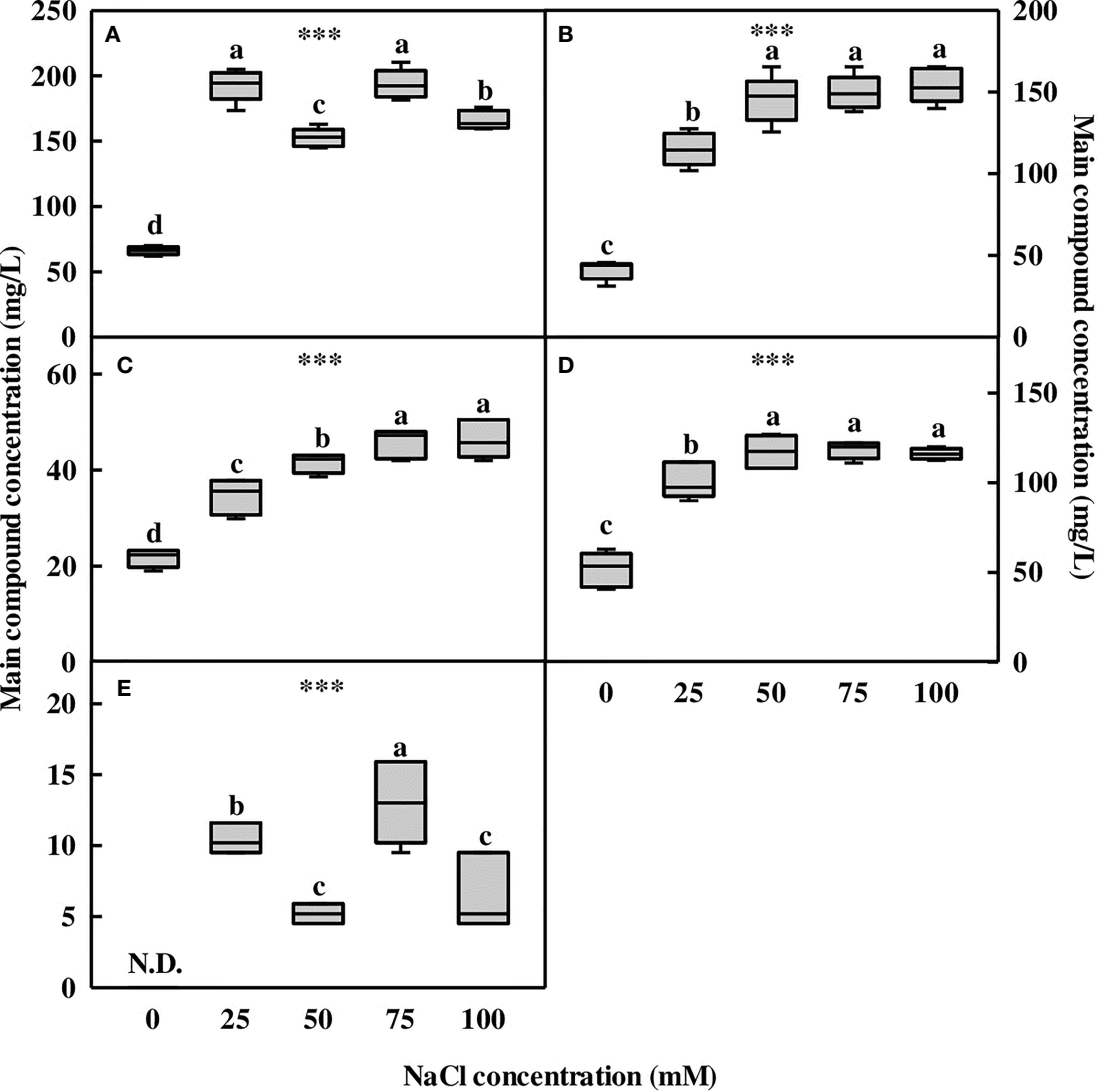
The concentration of the major compounds of *Limonium tetragonum* under different NaCl concentrations analyzed using LC-ESI-MS (*n* = 5). **(A)** Compound **1** (myricetin-3-O-β-D-galactoside), **(B)** compound **2**, **(C)** compound **3**, **(D)** compound **4**, and **(E)** compound **5**. Statistical analysis was performed with Duncan’s multiple range test. ***p = 0.001. N.D., non-detected.

The five compounds (**1–5**) contained in *L. tetragonum* were measured *via* liquid chromatography-electrospray ionization-mass spectrometry high-performance liquid chromatography (LC-ESI-MS HPLC). The metabolite concentration accumulated in the aerial part of *L. tetragonum* was significantly increased by NaCl treatment (**Figure 3**). Myricetin-3-O-β-D-galactoside (compound **1**) was found to be 66.2 ppm in the 0-mM NaCl, and it increased in response to NaCl treatment (**Figure 3A**). The concentration of “compound **1**” was higher at 25 mM (192.8 ppm) and 75 mM (193.7 ppm) of NaCl compared with the 0-mM NaCl. Among the NaCl treatments, 50- and 100-mM NaCl tended to decrease the “compound **1**” content compared with the other NaCl treatments. Myricetin-3-O-(3″-O-galloyl)-α-L-rhamnoside (compound **2**) gradually increased with NaCl concentration. The lowest concentration (41 ppm) was observed in the 0-mM NaCl, and it significantly increased in all NaCl treatments (**Figure 3B**). The concentration of “compound **2**” was the highest at 100-mM NaCl (154.1 ppm), but there was no significant difference between 50- and 75-mM NaCl, and these concentrations increased by 254.1%–275.8% compared with the 0-mM NaCl. Although 25-mM NaCl (115 ppm) showed the lowest value among the NaCl treatments, it increased by 180.4% compared with the 0-mM NaCl. Myricetin-3-O-α-L-rhamnoside (compound **3**) gradually increased with NaCl concentration (**Figure 3C**). Compound **3** in the 0-mM NaCl (21.66 ppm) was the lowest, and it significantly increased in all NaCl treatments compared with the 0-mM NaCl. It was the highest at 100 mM of NaCl, but there was no significant difference compared with 75-mM NaCl, and it increased by 114.4% and 110.4%, respectively, compared with the 0-mM NaCl. Myricetin-3-O-(2″-O-galloyl)-α-L-rhamnoside (compound **4**) was found to be 51.6 ppm in the 0-mM NaCl and was increased in all NaCl treatments compared with the 0-mM NaCl (**Figure 3D**). A tendency to increase at NaCl concentrations above 50 mM was observed, but there was no significant difference among the treatments. The concentration of “compound **4**” was the lowest at 25-mM NaCl compared with the other NaCl treatments but increased by 95.8% compared with the 0-mM NaCl. 5,7,2′,5′-Tetrahydroxy flavanone-3-O-β-D-galactoside (compound **5**) was not detected in the 0-mM NaCl but was found only in small amounts in the NaCl treatment (**Figure 3E**). At 75-mM NaCl (13ppm), it showed the highest value among the other NaCl treatments. It was found that the concentration of “compound **5**” was the lowest at 50- and 100-mM NaCl.

Myricetin derivatives and their glycosides were identified as the major compounds contained in *L. tetragonum* (Lee et al., 2011). Of the five major flavonoids in *L. tetragonum*, the concentration of myricetin glycosides (**1–4**) was increased in the NaCl treatment, and the flavanone compound (**5**) was detected only in the NaCl treatments. In addition, the concentration of the five compounds increased to the maximum in the 75-mM NaCl treatment than in the 0-mM NaCl treatment. Myricetin glycosides and flavanone compounds are in the flavonoid family and are the most abundant substances in *L. tetragonum*. Flavonoids and Pro are essential protective substances with antioxidative properties (Zhu, 2001). Many studies have reported that salt stress affects the flavonoid biosynthetic pathway in plants, leading to an increase in flavonoids (Ben Abdallah et al., 2016). Based on the above results, it can be inferred that the flavonoid biosynthetic pathway is stimulated by salt stress.

### 3.4 Transcriptome analysis

#### 3.4.1 Differentially expressed genes

**Figure 4:**
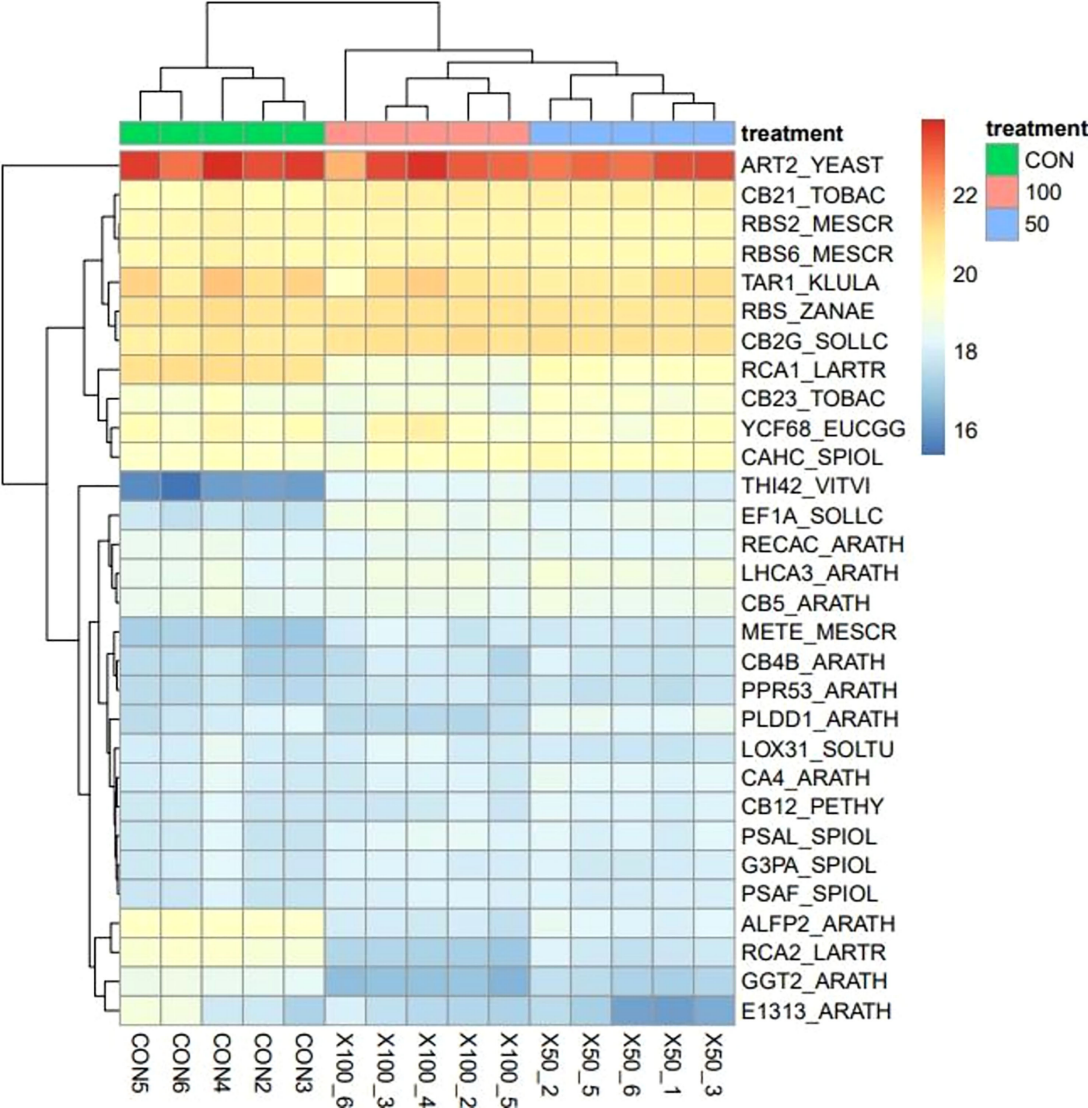
Heatmap using hierarchical clustering analysis for the top 30 most expressed genes.

The difference in the transcriptional characteristics expressed in the leaves of *L. tetragonum* in response to the NaCl concentration is shown in **Table 4** and **Figures 4** **–** **6**. Hierarchical clustering showed a clear separation between groups, and the principal component analysis (PCA) plots suggest that each sample was inherently different from the NaCl non-treated sample.

**Table 4:** Differential expression of genes related to the KEGG pathway in the NaCl concentrations.

Gene symbol	Entrez Gene ID	Description	Fold change (log_2_ ratio/0 mM of NaCl)	
			50 mM	100 mM
Biosynthesis of secondary metabolites				
*SDR1*	825294	NAD(P)-binding Rossmann-fold superfamily protein	2.25	5.52
*ASE2*	829626	GLN phosphoribosyl pyrophosphate amidotransferase 2	−3.30	−2.49
*CAT2*	829661	Catalase-2	2.50	2.74
*AT2G26800*	817221	Aldolase superfamily protein	2.03	3.97
*PRX52*	830416	Peroxidase superfamily protein	2.13	−4.15
*VTC2*	828792	GDP-L-galactose phosphorylase 1	4.21	2.75
*NCED4*	827655	Nine-cis-epoxycarotenoid dioxygenase 4	−3.10	3.45
*OMT1*	835504	O-methyltransferase 1	2.40	3.16
*NPC1*	837234	Non-specific phospholipase C1	2.10	2.84
Tryptophan metabolism				
*YUC10*	841313	Flavin-containing monooxygenase family protein	2.13	−2.59
*LOC110894758*	110894758	Catalase-like	2.50	2.74
*OMT1*	835504	O-methyltransferase 1	2.40	3.16
Circadian rhythm: plant				
*LHY*	839341	Homeodomain-like superfamily protein	7.20	3.18
*ELF3*	1999	E74 like ETS transcription factor 3	−2.17	2.04
*PRR5*	832518	Two-component response regulator-like protein	−5.83	2.12

Values indicate log2 ratios obtained based on the RNA-seq data. Calculations based on NaCl concentrations of five replicates.

To analyze the reliable data, noise was excluded (signal evaluation).

Hierarchical clustering using heatmaps revealed differences between the 0-mM NaCl and the 50- and 100-mM NaCl treatments (**Figure 4**). The *THI42_VITVI* (TV) was upregulated in the NaCl treatment compared with the 0-mM NaCl. The expression of genes in plants changes under the influence of salinity, and their expression profiles have been studied in different plant species and at different stages of development in response to salinity stress (Jamil et al., 2011). The transcriptome analysis revealed differences in gene expression between the *L. tetragonum* NaCl treatment and the 0-mM NaCl. Some major salt-related components and DEGs were identified. The results of the present study can be used as clues for the analysis of the salinity response and salinity resistance in *L. tetragonum*.

TV, a protein-coding factor, is a thiamine thiazole synthase and was upregulated by NaCl treatment. Thiamine is an essential water-soluble vitamin for all organisms, found in high amounts in green vegetables, beans, cereal germs, and nuts (Goyer, 2010). NaCl treatment of the shiitake plant has the potential to enhance its thiamine content.

#### 3.4.2 Principal component analysis

**Figure 5:**
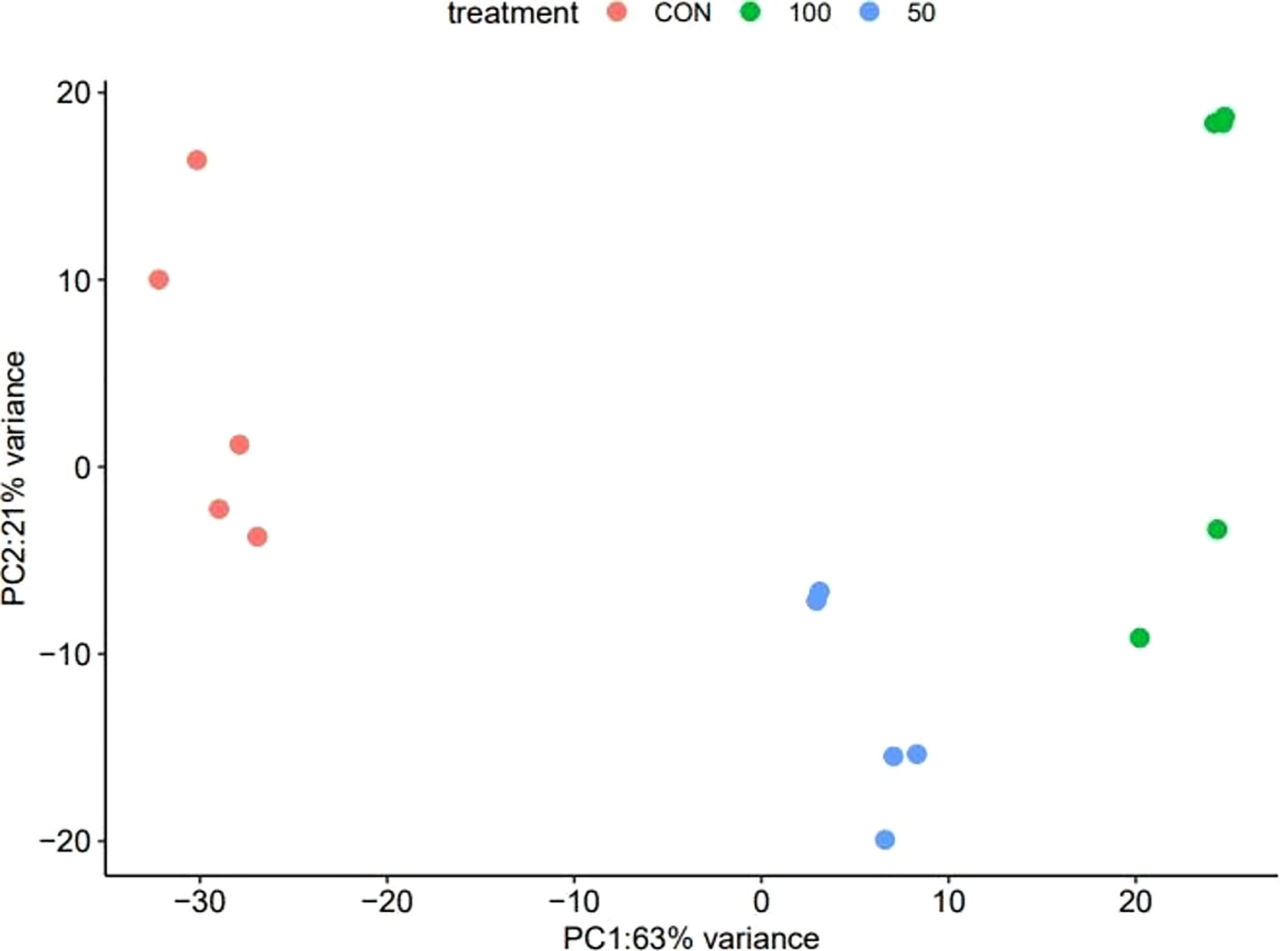
The principal component analysis of *Limonium tetragonum* under different NaCl concentrations (*n* = 5).

The PCA revealed that the 0-mM NaCl and the NaCl treatments were clearly distinguished (**Figure 5**). In addition to the difference between the 0-mM NaCl and the NaCl treatments, the dispersion distribution was displayed in accordance with the NaCl concentration, suggesting that the greater the NaCl concentration, the greater the differential expression in *L. tetragonum*.

#### 3.4.3 Gene ontology

**Figure 6:**
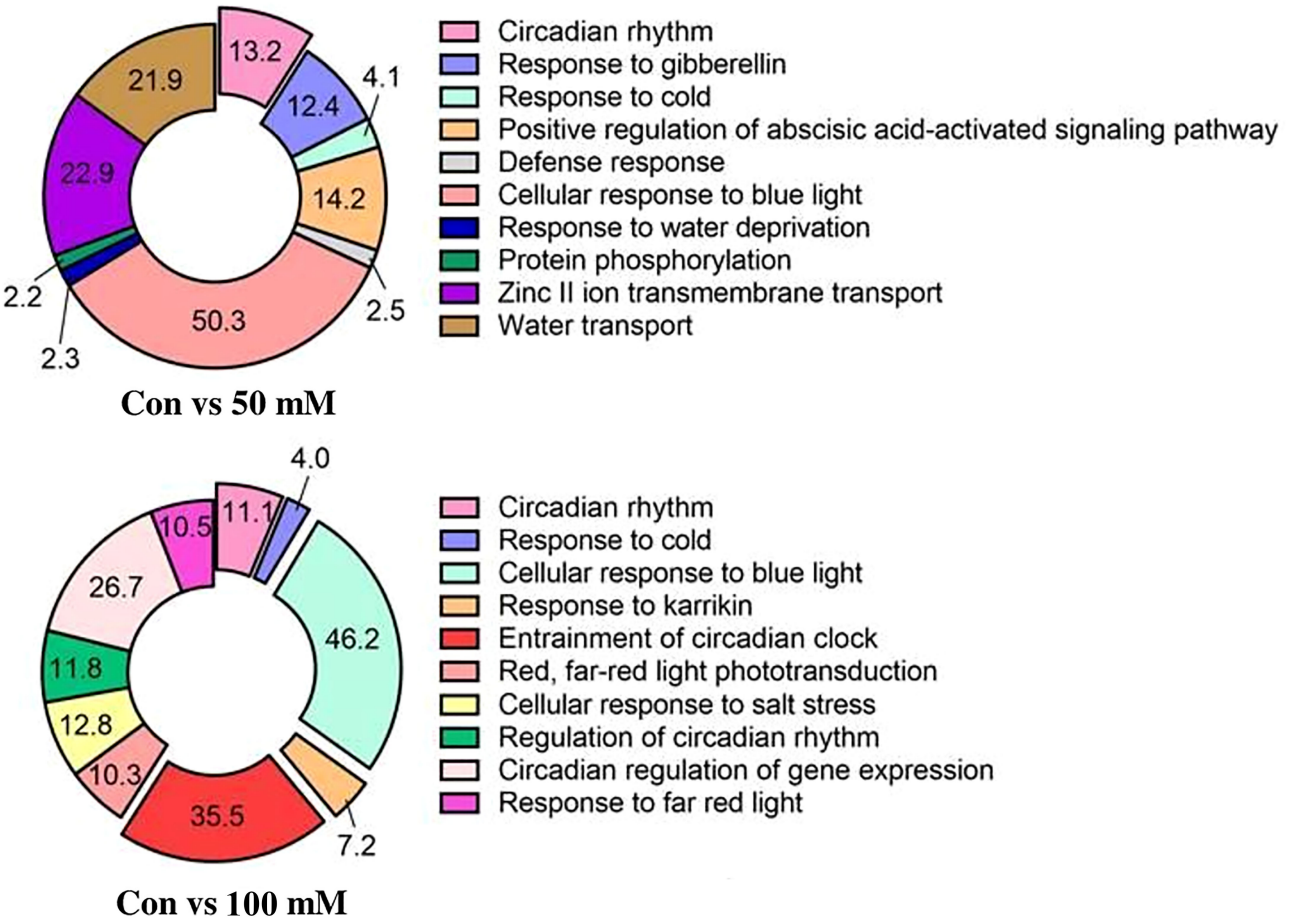
Gene ontology analysis: biological processes for NaCl concentrations (*n* = 5).

The biological processes of *L. tetragonum* were changed according to the NaCl concentration (**Figure 6**). The most abundant GO associated with biological processes in the 50-mM NaCl/0-mM NaCl was circadian rhythm (50%). Circadian rhythms are a subset of biological rhythms with cycles defined as the time to complete one circadian cycle (Dunlap and Loros, 2004). The transcription rate and transcriptional accumulation of *Arabidopsis LHCB* (Millar and Kay, 1991) and many other genes (McClung and Kay, 1994) are associated with circadian regulation (McClung, 2006). Even in the 100-mM NaCl/0-mM NaCl, circadian rhythms accounted for a large portion and may be a major contributor to salt tolerance.

#### 3.4.4 KEGG pathway

The KEGG analysis revealed common pathways, viz., “biosynthesis of secondary metabolites,” “tryptophan metabolism,” and “circadian rhythm: plant” in the 0-mM NaCl and the 50- and 100-mM NaCl (**Table 4**). There were no significantly enriched molecular function terms in DEG, but the 100-mM NaCl enriched several cellular component terms, i.e., the chloroplast and plasma membrane. The KEGG pathway analysis results were similar to the GO “biological processes” terms. At 50-mM NaCl, “tryptophan metabolism” was found to be the most enriched pathway, followed by “circadian rhythm-plant,” “MAPK signaling pathway-plant,” and “biosynthesis of secondary metabolites.” However, “biosynthesis of secondary metabolites” and “tryptophan metabolism” were not statistically significant pathways at 100-mM NaCl.

The genes involved in secondary metabolite biosynthesis pathways are shown in **Table 4**. *Serine dehydratase regulator 1* (*SDR1*), *dominant cataract 2* (*CAT2*), *Aldolase superfamily protein* (*AT2G26800*), *GDP-L-galactose phosphorylase 1* (*VTC2*), *O-methyltransferase 1* (*OMT1*), and *NPC intracellular cholesterol transporter 1* (*NPC1*) are genes that are commonly upregulated in the NaCl treatments compared to the 0-mM NaCl. *GLN* phosphoribosyl pyrophosphate amidotransferase 2 (*ASE2*) was downregulated in the NaCl treatment compared with the 0-mM NaCl. Specifically, *peroxidase superfamily protein* (*PRX52*) and *nine-cis-epoxycarotenoid dioxygenase 4* (*NCED4*) showed differential expression patterns according to the NaCl concentration. *PRX52* was upregulated compared with the 0-mM NaCl at 50-mM NaCl but downregulated at higher NaCl concentrations. *NCED4* was downregulated at 50-mM NaCl but upregulated at higher NaCl concentrations.

*SDR1* is a gene involved in sugar sensing and signaling essential for many metabolic processes such as germination and growth in *Arabidopsis* (Rolland et al., 2002). *SDR1* was found to be upregulated with increasing NaCl concentration. CAT catalyzes the breakdown of H_2_O_2_ into water and oxygen (Willekens et al., 1997). CAT expression/activity was found to be higher in halophytes than in glycophytes during salinity stress, suggesting that CAT helps in salt tolerance by scavenging H_2_O_2_ (Bose et al., 2014). In halophytes, salt stress increases the production of the phenylpropanoid genes *PRX52* and *OMT1* (Gao et al., 2019).

Genes belonging to the tryptophan metabolism pathway were *flavin-containing monooxygenase family protein* (*YUC10*), *catalase-like* (*LOC110894758*), and *OMT1*. The relative expression of *YUC10* was upregulated at 50 mM of NaCl but downregulated at 100 mM of NaCl. *LOC110894758* and *OMT1* were upregulated in the NaCl treatment. *OMT1* was commonly observed in secondary metabolite biosynthesis and tryptophan metabolism.

The *YUC* gene induces the biosynthesis of auxin and is a developmental induction gene necessary for the development of embryogenesis, seedlings, and flowers. *YUC10* is an essential gene for the embryogenesis development and leaf formation of *Arabidopsis* and induces plant development (Cheng et al., 2007). In the leaves of *L. tetragonum* grown with 50-mM NaCl, *YUC10* was upregulated, indicating its role in the growth and development of *L. tetragonum* in a saline environment. The KEGG pathway analysis revealed that “biosynthesis of secondary metabolites” and “tryptophan metabolism” were consistently enriched pathways in the database, requiring further investigation with respect to secondary metabolites present in *L. tetragonum*.

The protein–protein interaction network analysis using the STRING database showed similar results as the KEGG pathway analysis. The molecules related to the “circadian rhythm” are well clustered in the network. The circadian rhythm, which accounted for the largest proportion in the enriched GO term, was upregulated in the NaCl treatment, and a significant difference was observed at different NaCl concentrations. Homeodomain-like superfamily protein (*LHY*) was upregulated in the NaCl treatment, with the highest expression at 50 mM of NaCl. E74-like ETS transcription factor 3 (*ELF3*) and *PRR5* (proline-rich 5) were downregulated at 50-mM NaCl but upregulated at 100-mM NaCl.

*LHY* is one of the *Arabidopsis* homologs and is a gene involved in flowering, stomatal development, and stress regulation in the succulent *Suaeda fruticosa* (Diray-Arce et al., 2019). *Arabidopsis Early Flowing 3* (*AELF3*) is a gene associated with a circadian clock that physically interacts with photoreceptors (Liu et al., 2001) and induces proper expression of plant growth promoters in *Arabidopsis* (Nusinow et al., 2011).

## 4 Conclusion

Our results showed the same results as the studies on the NaCl concentration of *L. tetragonum.* NaCl treatment at less than 100 mM in a hydroponics system did not affect the growth and Chl fluorescence of *L. tetragonum*, indicating that *L. tetragonum* was tolerant to the saline environment. The water potential of the leaves of *L. tetragonum* decreased as NaCl concentration increased, indicating that solutes accumulated in the leaves increased. The leaves of *Limonium tetragonum* accumulated minerals and Na^+^ was found to have the highest concentration. The K^+^ content decreased in inverse proportion to the Na^+^. NaCl treatment increased some of the AAs of the leaves of *L. tetragonum*, and among them, the proline content increased significantly. The chemical profiling was done using LC-ESI-MS HPLC. In this study, the concentration of the four myricetin glycosides and the flavanone compound was significantly increased by NaCl treatment. Flavanone was found in the form of 5,7,2′,5′-tetrahydroxy flavanone-3-O-β-D-galactoside in *L. tetragonum*, and its content improved in all the NaCl treatments. Transcriptome analysis of the aerial part of *L. tetragonum* showed clear differences in the secondary metabolite biosynthetic pathway, tryptophan metabolome, and circadian rhythm between the 0-mM NaCl and the NaCl treatments. The NaCl concentrations of 100 mM or less did not affect the growth of *L. tetragonum*. However, a number of DEGs identified in the transcriptome analysis of plants grown in NaCl treatment confirmed differences in the regulatory machinery under NaCl conditions. This study describes a methodology to improve the functional materials in the NaCl range that do not reduce the growth of *L. tetragonum* in vertical farms. In addition, this study revealed that NaCl treatment was necessary to improve the flavonoid myricetin glycoside. The optimal NaCl concentration for the enhancement of the secondary metabolites of *L. tetragonum* in the present study is 75-mM NaCl.

## Data availability statement

The original contributions presented in the study are publicly available. This data can be found here: https://www.ncbi.nlm.nih.gov/bioproject/929564.

## Author contributions

K-HS and EJ designed the experiments. K-HS, KC, and JY organized the project. S-NJ, M-JK, and YK performed the experiments. S-NJ wrote the manuscript. All authors contributed to the article and approved the submitted version.

## Supplementary material

Supplementary Figure 1 The photon flux density for the wavelength of the LED.

Supplementary Figure 2 The growth of *L. tetragonum* under different NaCl concentrations.

Supplementary Table 1 Composition of Hoagland’s nutrient solution used in the cultivation of *Limonium tetragonum*.

Supplementary Table 2 NaCl components and chemical property for *L. tetragonum* under different NaCl concentrations used in this study.
