# Supplementary figures and images for: Physiological and biochemical responses of Limonium tetragonum to NaCl concentrations in hydroponic solution
Source: Front Plant Sci. 2023 Apr 26;14:1159625. doi: 10.3389/fpls.2023.1159625 (PMC10170659; doi:10.3389/fpls.2023.1159625)

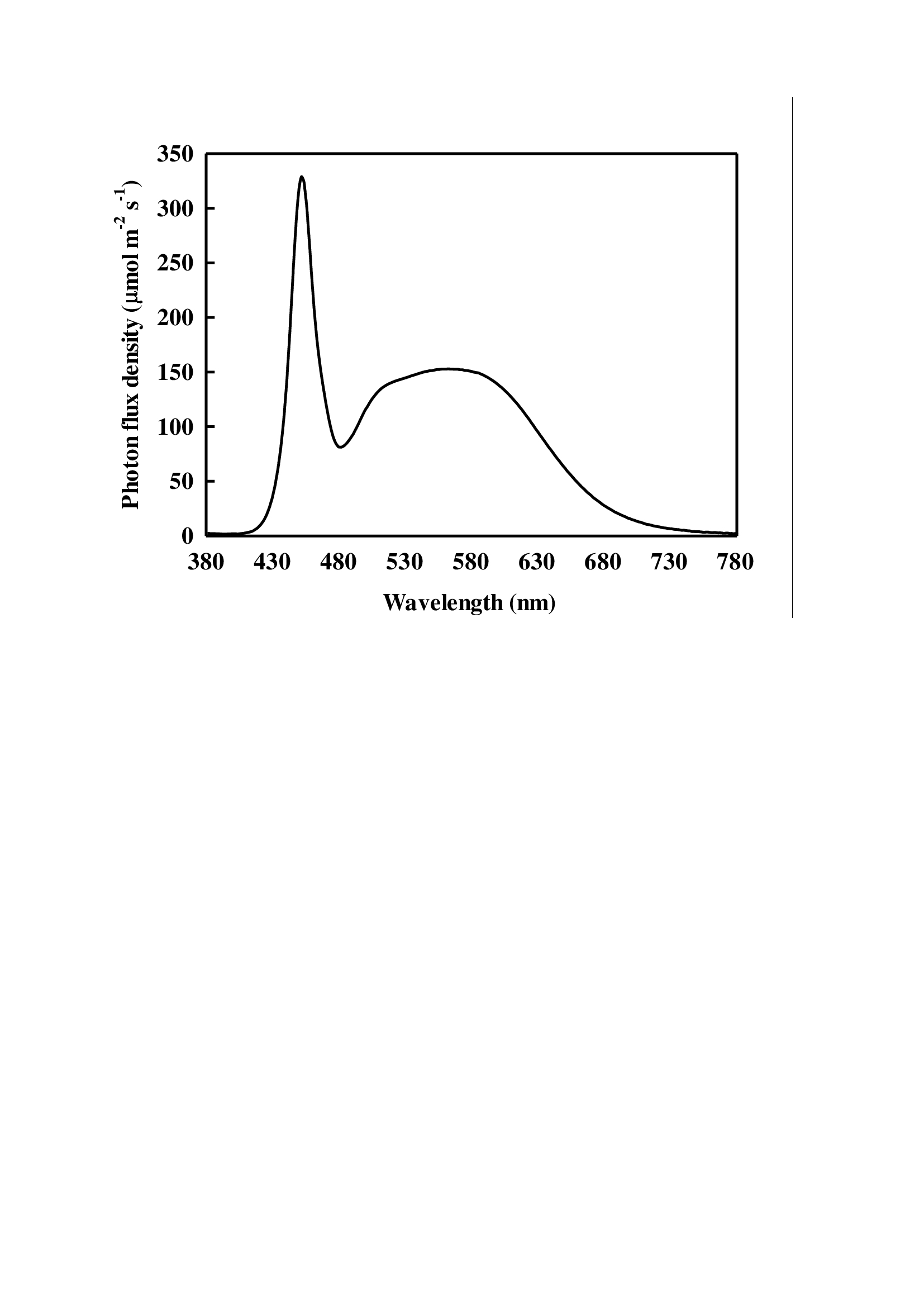

Supplement: Supplementary Figure 1 — The photon flux density for the wavelength of the LED. [file Image_1.jpeg]

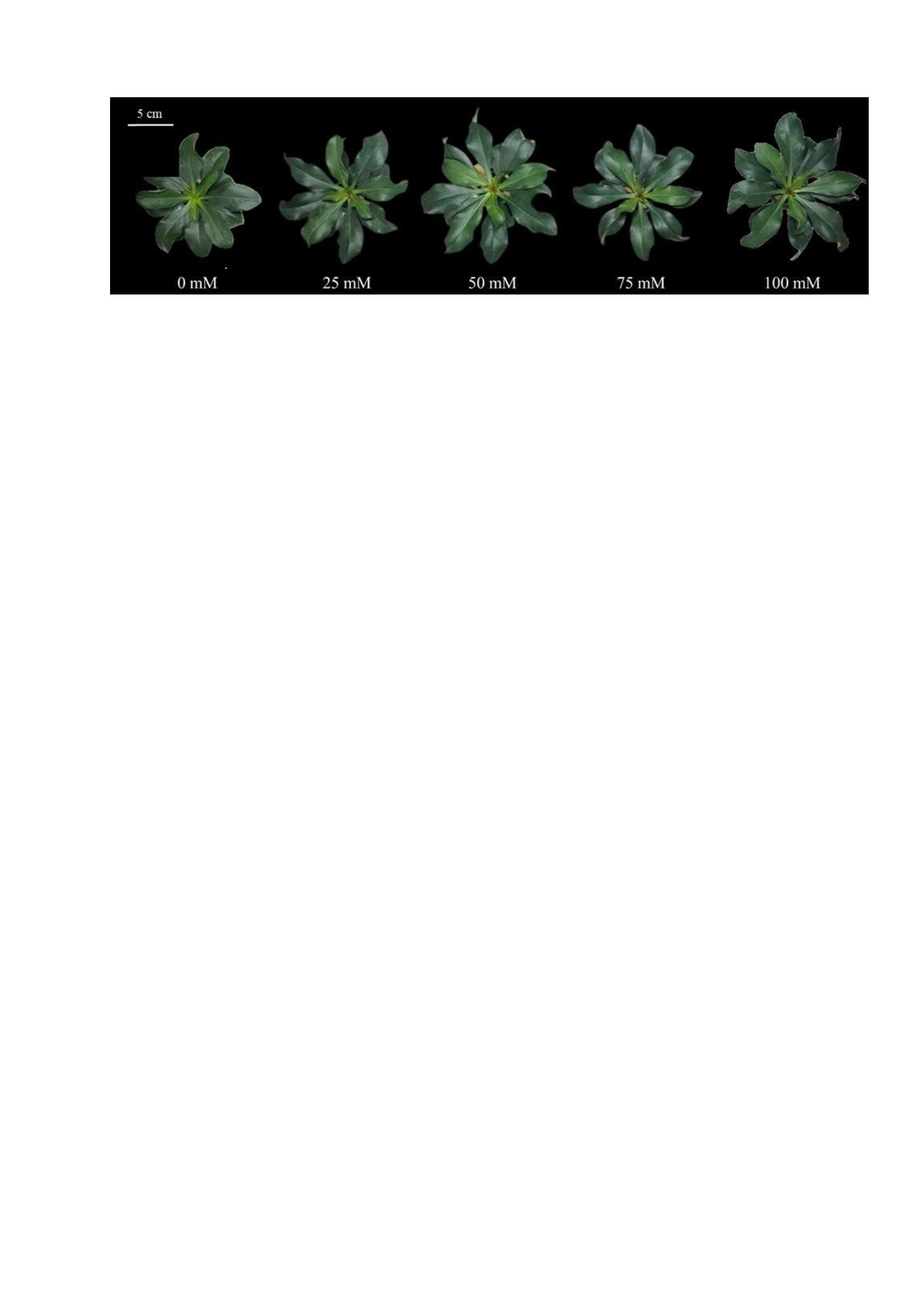

Supplement: Supplementary Figure 2 — The growth of L. tetragonum under different NaCl concentrations. [file Image_2.jpeg]
