# Supplementary material for: Physiological and biochemical responses of Limonium tetragonum to NaCl concentrations in hydroponic solution
Source: Front Plant Sci. 2023 Apr 26;14:1159625. doi: 10.3389/fpls.2023.1159625 (PMC10170659; doi:10.3389/fpls.2023.1159625)
Supplement: Supplementary Table 1 — Composition of Hoagland’s nutrient solution used in the cultivation of Limonium tetragonum. [file Table_1.docx]

**Supplementary Table S1. Composition of Hoagland's nutrient solution used in the cultivation of *Limonium tetragonum.***

| Type | Chemical | Amount (g/L) |
| --- | --- | --- |
| A | KNO^3^ | 236.15 |
|  | Ca(NO^3^)^2^·4H^2^O | 75.825 |
|  | Fe-EDTA | 10.5275 |
| B | KNO^3^ | 75.825 |
|  | NH^4^H^2^PO^4^ | 28.765 |
|  | MgSO^4^·7H^2^O | 123.24 |
|  | H^3^BO^3^ | 0.3575 |
|  | MnSO^4^·4H^2^O | 0.22625 |
|  | ZnSO^4^·7H^2^O | 0.0275 |
|  | CuSO^4^·5H^2^O | 0.01 |
|  | Na^2^MoO^4^·2H^2^O | 0.0025 |
|  | Total | 550.9563 |
