# Supplementary material for: Physiological and biochemical responses of Limonium tetragonum to NaCl concentrations in hydroponic solution
Source: Front Plant Sci. 2023 Apr 26;14:1159625. doi: 10.3389/fpls.2023.1159625 (PMC10170659; doi:10.3389/fpls.2023.1159625)
Supplement: Supplementary Table 2 — NaCl components and chemical property for L. tetragonum under different NaCl concentrations used in this study. [file Table_2.docx]

**Supplementary Table S2. NaCl components and chemical property for *Limonium tetragonum* under different NaCl concentrations used in this study.**

| NaCl concentrations (mM) | Chemical | Amount (g/L) | Electrical conductivity (dS/cm) |
| --- | --- | --- | --- |
| 0 | NaCl | - | 1.121 |
| 25 |  | 1.461 | 3.960 |
| 50 |  | 2.922 | 6.520 |
| 75 |  | 4.383 | 9.420 |
| 100 |  | 5.844 | 11.620 |
